# Epileptic seizures triggered by eating in dogs

**DOI:** 10.1111/jvim.15773

**Published:** 2020-04-28

**Authors:** Josep Brocal, Mark Lowrie, Gemma Wamsley, Alberto Cauduro, Paul Mandigers, Rodrigo Gutierrez‐Quintana, Catherine Stalin

**Affiliations:** ^1^ Wear Referrals Veterinary Hospital Stockton‐on‐Tees UK; ^2^ Dovecote Veterinary Hospital Derby UK; ^3^ Department of Musculoskeletal Biology Institute of Ageing and Chronic Disease, University of Liverpool Neston UK; ^4^ Associazione Professionale Neurovet Legnano Italy; ^5^ Department of Clinical Sciences of Companion Animals Utrecht University Utrecht The Netherlands; ^6^ School of Veterinary Medicine, College of Medical, Veterinary and Life Sciences, University of Glasgow Glasgow UK

**Keywords:** drinking, food, reflex, seizures, stimuli

## Abstract

**Background:**

Seizures triggered by eating (STE) behavior are very rare in humans and have not been documented previously in dogs.

**Objectives:**

To document the occurrence of STE in dogs and describe their clinical features.

**Animals:**

Ten client‐owned dogs with STE diagnosed at 5 European referral centers.

**Methods:**

A call for suspected cases of STE was made online. This call was followed by a retrospective review of medical records, combined with a questionnaire to be completed by both the owner and the board‐certified neurologist who made the diagnosis. Cases were included if >50% of the seizures that occurred were related to eating and if a minimum diagnostic evaluation for seizures had been performed.

**Results:**

Four cases only had STE and 6 cases had both STE and spontaneous seizures. Four of the dogs were retrievers. The most common seizure type was focal epileptic seizures evolving to become generalized. Nine dogs were diagnosed with idiopathic epilepsy. One dog had a presumptive diagnosis of glioma involving the margins of the parietal, temporal, and frontal cortex (the perisylvian region), an area known to have a key role in eating‐associated epilepsy in people. Treatment strategies included a combination of pharmacological management and eating habit changes.

**Conclusions and Clinical Importance:**

We have identified a form of reflex epilepsy in dogs, with STE behavior. Further studies are warranted to improve the characterization and management of STE.

**Abbreviations**
CRI: constant rate infusion
CT: computed tomography
EEG: electroencephalography
FLAIR: fluid‐attenuation inversion recovery
GRE: gradient recalled echo
IE: idiopathic epilepsy
IVETF: International Veterinary Epilepsy Task Force
MRI: magnetic resonance imaging
SRS: spontaneous recurrent seizures
STE: seizures triggered by “eating”

## 1 INTRODUCTION

Reflex epilepsy is defined by the International Veterinary Epilepsy Task Force (IVETF) as epileptic seizures that are objectively and consistently evoked by a specific afferent stimulus or by activity of the patient.^1^ Eating is an example of several potential triggers.^2^

In humans, a variety of sensory, motor, and cognitive functions can trigger reflex seizures. Visual, auditory, somatic sensory, and proprioceptive stimuli associated with seizures include flashing lights, music, hot water, and startling. Seizures also may be induced by thinking or performing complex cognition‐guided tasks (praxis; eg, writing).^2^ Reflex seizures are uncommon in dogs, but some reports do exist. Light, sound, and movement in the visual field are well‐known triggers for myoclonic epilepsy in dogs with Lafora disease.^3^, ^4^ Intense physical activity was associated with seizures in 1 dog, and proprioceptive stimulation could have been the trigger.^5^ Visits to a specific location, having visitors at home, a change in the life situation, a change in the daily routine, altered sleep patterns, unfamiliar places, weather, and estrus have been identified as triggering stimuli or precipitating factors for seizures in dogs.^6^, ^7^ The difference between nonreflex precipitated seizures and reflex seizures is unclear and the human medical literature has been inconsistent.^8^ A conceptual continuum might exist between reflex and spontaneous seizures. Distinction between reflex and nonreflex precipitated seizures might be meaningful, and specifying with as much detail as possible the phenomena observed is recommended.^8^

Eating epilepsy is characterized by seizures closely related to eating behavior, which occur in patients with or without spontaneous recurrent seizures (SRS). Seizures triggered by eating (STE) are very rare, and reports in affected humans are sparse. The estimated prevalence is only 1 per 1000 to 2000 of all human patients with epilepsy,^9^ and 7 per 1000 patients with epilepsy refractory to medical management.^10^ It is a unique form of epilepsy frequently classified as reflex with discrete electroencephalographic and clinical signs, and pathophysiological mechanisms^10^, ^11^ that has not been reported previously in dogs. The purpose of our study was to document the occurrence of STE in dogs, describe their clinical features and their response to treatment. Our hypothesis was that, although rare, STE do occur in dogs and can have variable semiology and pathophysiology.

## 2 MATERIALS AND METHODS

Cases were recruited using an online veterinary forum (Veterinary Information Network‐VIN American College of Veterinary Internal Medicine‐ACVIM/European College of Veterinary Neurology‐ECVN Neurologists ListServe) asking for veterinarians to contact us with cases of suspected STE behavior in dogs. A detailed questionnaire (Supplemental Questionnaire) to be completed by both veterinarians and owners by telephone interview or online was designed. The questionnaire included mainly closed‐ended and multiple‐choice questions with several open‐ended questions where more specific details were requested. Questions aimed to provide phenotypic information in terms of the signalment of the animals, seizure semiology, results of diagnostic investigations, and treatment. The questions also aimed to investigate precipitating factors previously investigated in humans with STE. Questions regarding the characteristics of the episodes included an opportunity to provide a free‐text description. If >1 type of seizure or episode was displayed by a patient, owners were asked to describe each separately. Video recordings of the episodes where available were reviewed by an investigator to confirm that the episodes appeared to represent an epileptic seizure and to improve the phenotypic characterization provided by owner or veterinarian. Inclusion criteria were: episodes classified as STE by 2 board‐certified neurologists (J. B. and the board‐certified neurologist from the initial referral institution), a minimum of 3 STE; >50% of the seizures occurred in relation to eating during at least 3 months, and a minimum database of investigations for seizures including hematology, serum biochemistry and magnetic resonance imaging (MRI) of the head.

We adhered to terminology and classification proposed by the IVETF whereby cluster seizures are defined clinically as ≥2 seizures within a 24‐hour period, and status epilepticus is defined as >5 minutes of continuous epileptic seizures or ≥2 discrete epileptic seizures between which there is incomplete recovery of consciousness (for generalized convulsive seizures).^1^ Descriptive statistics are reported as median and range.

## 3 RESULTS

### 3.1 Animals

Eleven cases were identified from 5 European referral institutions. Ten dogs met the inclusion criteria (1 case was excluded because advanced imaging had not been performed). Breeds represented included the Golden Retriever (n = 2), Bichon Frise (n = 1), Jack Russell Terrier (n = 1), Poodle (n = 1), English Staffordshire Bullterrier (n = 1), Border Collie (n = 1), Border Terrier (n = 1), Curly‐Coated Retriever (n = 1), and Flat‐Coated Retriever (n = 1) (Supplemental Table S1). Six dogs were female (1 intact and 5 spayed) and 4 were male (3 intact and 1 neutered; Supplemental Table S1). One female was in estrus at seizure onset and was spayed afterward. The median weight at seizure onset was 17 kg (range, 6‐37 kg).

None of the owners had experienced difficulty in training their dogs, and all dogs were reported to be normal between seizures. Five of the dogs lived with other dogs. Five owners knew some of their dogs' siblings and the male sibling of 1 Golden Retriever had SRS. Six dogs were known to have uncomplicated births and in 4 dogs it was unknown if they had been born by Caesarean section. None had a history of head trauma. Three dogs occasionally had diarrhea associated with scavenging or sharing food, and no dogs were reported to vomit regularly.

### 3.2 Seizure characteristics

The median age at STE onset was 32 months (range, 12‐120 months) and the median age at SRS onset was 27 months (range, 12‐42 months). Excluding 1 dog (with structural epilepsy and age at onset of 120 months) all dogs were <6 years of age at STE onset (median, 27 months; range, 12‐69 months; Supplemental Table S1).

Video recordings of STE were provided for 8 dogs (Supplemental Videos S1 and S2). Four dogs had STE and 6 dogs had both STE and SRS. The first witnessed seizure was triggered by eating in 8 dogs and was spontaneous in 2 (Supplemental Table S1).

No pre‐ictal signs were reported. Seizures triggered by eating and SRS frequently started with facial or leg twitching (7/10), lifting of a limb (4/10), opening the mouth or jaw chattering (4/10), head turn (2/10), and vigorous head shaking (1/10). All but 1 dog frequently fell into lateral recumbency, and all dogs had tonic, clonic, or tonic‐clonic movements of the limbs. Autonomic signs during STE and SRS were observed in all dogs: salivation was the most common (10/10) followed by urination (6/10) and defecation (5/10). All dogs had focal epileptic seizures that evolved into generalized epileptic seizures. Focal epileptic seizures did not always become generalized. No dogs were reported to have atonic or absence seizures and 1 dog had myoclonic seizures in addition to focal epileptic seizures that evolved into generalized seizures. Seizures triggered by eating had the same semiology as the SRS in 5 of 6 dogs. Nine dogs had post‐ictal signs after both STE and SRS that lasted from 10 minutes to 2 hours (Supplemental Table S1).

According to inclusion criteria, STE represented >50% of all seizures witnessed during a 3‐month period. In dogs with both STE and SRS, STE were reported to represent 70% to 80% of all seizures consistently, except in 1 dog in which the percentage wax and waned, but frequently represented >50%.

Six dogs had cluster seizures. They occurred only once in 1 dog and twice in another (the dog with structural epilepsy). Cluster seizures were triggered only by eating in the dog with structural epilepsy and were both spontaneous and triggered by eating in the other dogs. One dog was unable to finish the same meal that triggered the first seizure and 2 owners usually did not allow their dogs to finish the meal to prevent further seizures. Status epilepticus was not reported in any dog.

### 3.3 Food‐related stimulus

Eight dogs were reported to be food oriented; 9 to have good appetite and 1 an average appetite. No recent change in diet had occurred for any dog. Both dry and canned food triggered STE in 8 dogs and the other 2 dogs ate only dry food. Food was always available for 2 dogs at onset of STE, with the reminder of the dogs being fed 2 to 4 times per day. Nine dogs frequently received food rewards, and they had triggered STE in 3, with STE occurring up to 50% of the times in 1 dog.

Most STE occurred at the beginning of meals (after a few bites; 6/10 dogs) and at the middle of meal (after eating more than 50% of the meal; 4/10 dogs); and, although reported, they occurred less frequently before starting a meal (ie, stimulated by visual stimulus and smell or when opening the mouth before eating) or after the end of a meal (when no food was left in the bowl and within 5 minutes of eating). The time of the meal was unrelated to occurrence of STE in 7 dogs, whereas 3 dogs were reported to have STE mostly at breakfast, breakfast and lunch, and breakfast and dinner. Excitement caused by getting food ready could precipitate seizures in 2 dogs. Drinking was reported to trigger seizures in only 1 dog that suffered from STE with no SRS, and in this case 50% of the STE were reported to be caused by drinking. The sight or smell of food, the size of the meal, altering the timing of the meal (eating late versus eating early), effect of food temperature (hot, room temperature or cold), and eating speed did not precipitate STE or the effects of these factors were unknown. It was difficult for owners to retrospectively evaluate the effect of some of these possible triggers on STE frequency. Owners could not identify any other triggers for seizures.

### 3.4 Investigations

**FIGURE 1:**
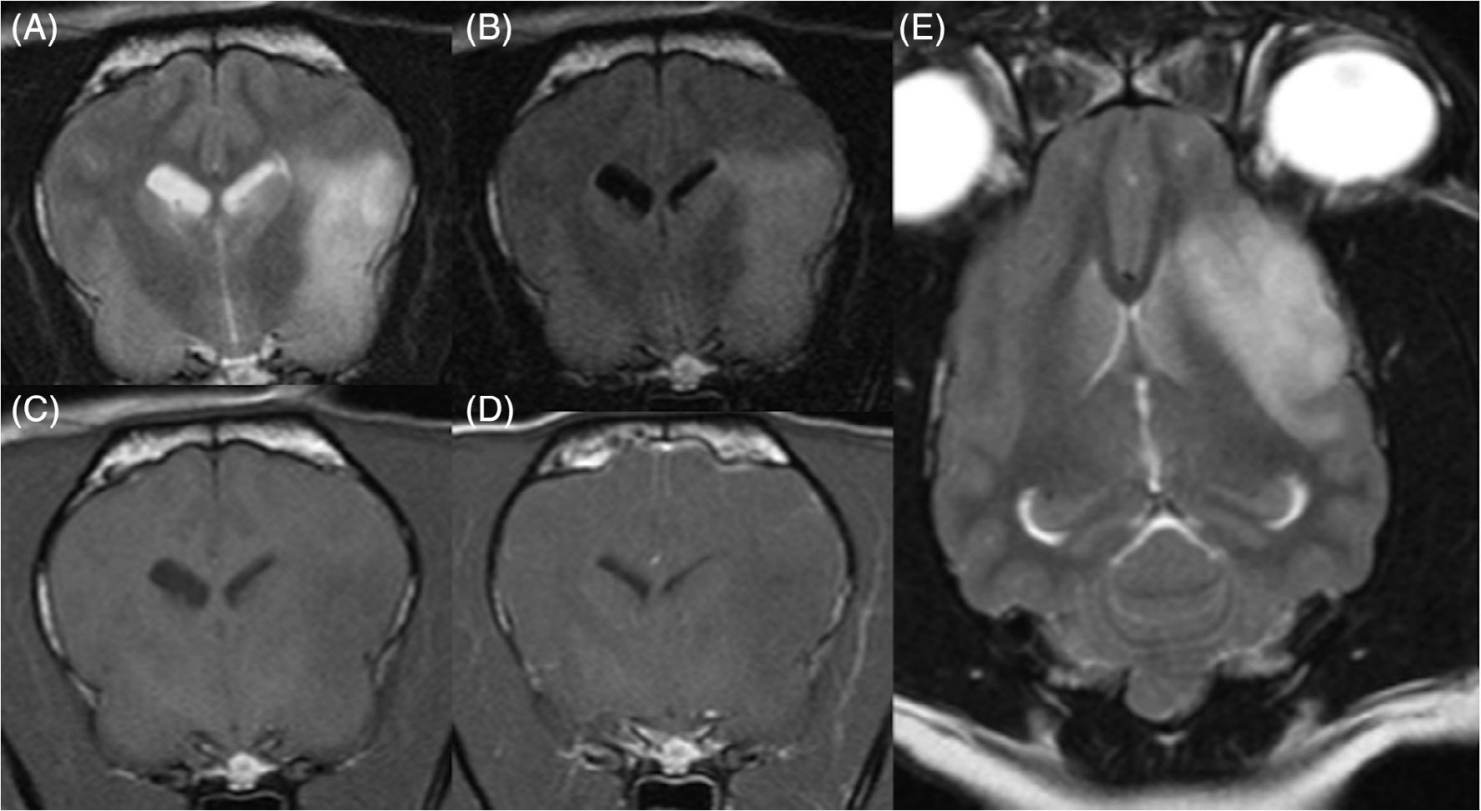
A, Transverse T2W, B, FLAIR, C, T1W, and D, T1W postcontrast; and dorsal T2W images of the dog with structural epilepsy (Case 10) illustrating the large ovoid‐shape ill‐defined intra‐axial homogeneously hyperintense on T2W and FLAIR images compared to grey matter, hypointense on T1W images, and with no contrast enhancement mass lesion affecting mainly the left temporal and frontal cortex causing mild mass effect

**FIGURE 2:**
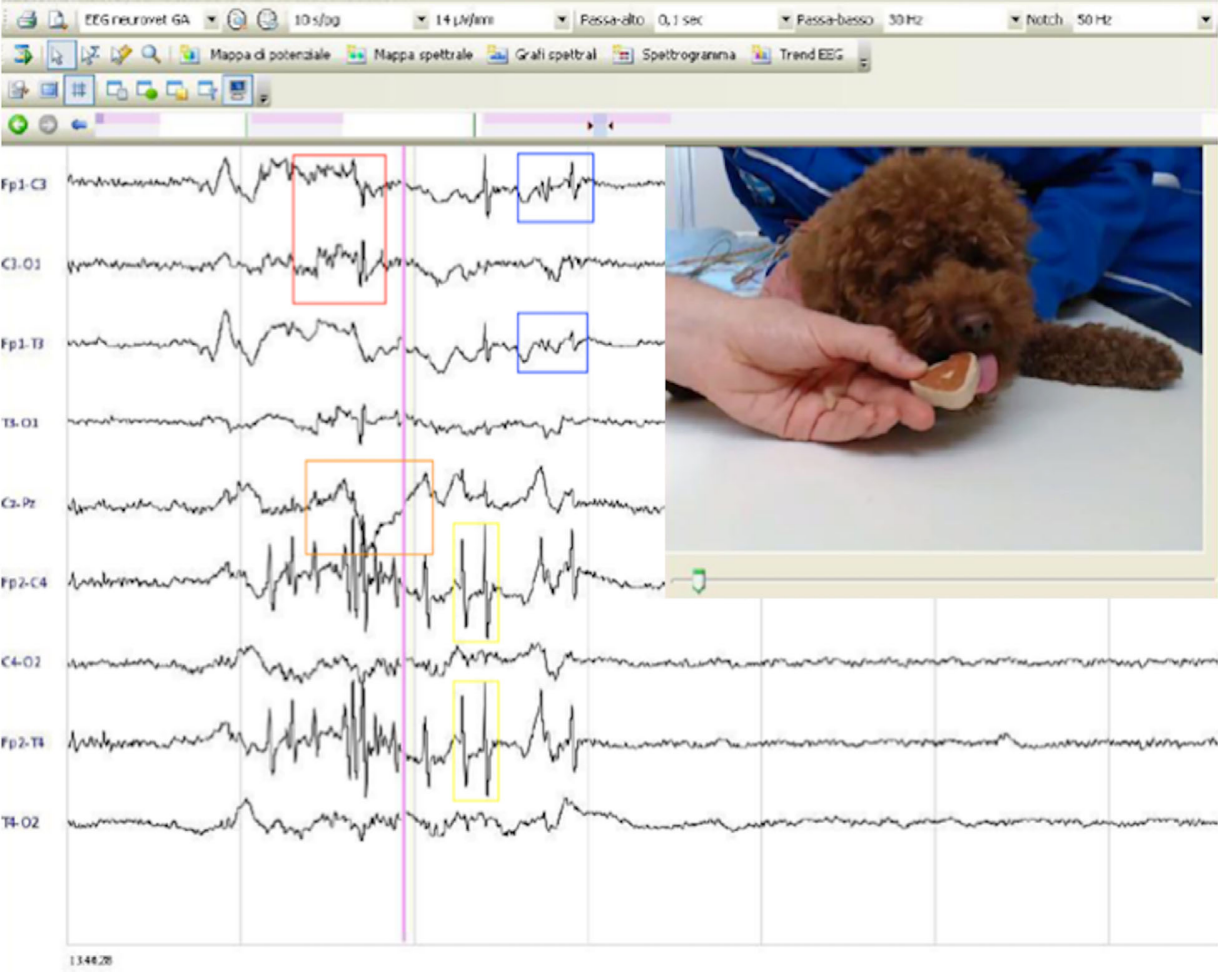
Unsedated eating video‐EEG of Case 6 illustrating muscular artifacts (red rectangle), blink reflex (yellow rectangles), eye movement (orange rectangle), and spikes (blue rectangles)

Physical and neurological examinations were normal in all but 1 dog. This dog had generalized ataxia and was slightly sedated because of antiepileptic treatment. Hematology, serum biochemistry, and serum electrolyte concentrations were normal in all dogs except for 1, which had increased alkaline phosphate activity secondary to treatment with phenobarbital. Serum ammonia concentrations in 3 dogs, bile acid stimulation tests in 7 dogs, resting bile acid concentration in 2 dogs, and urinalyses in 6 dogs all were normal. The dog with structural epilepsy had computed tomography (CT) of the thorax and abdominal ultrasound examination, both of which were normal. Only 1 other dog had an abdominal ultrasound examination, which also was normal. Magnetic resonance imaging of the head was performed using a high‐field magnet (1.5 Tesla) in 4 dogs and a low‐field magnet (0.25 and 0.27 Tesla) in 6 dogs. Magnetic resonance imaging was normal in 9 dogs and abnormal in 1 dog. This 10‐year‐old female neutered Border Terrier had a large ovoid‐shaped, ill‐defined, intra‐axial mass lesion affecting mainly the left temporal and frontal cortex, to a lesser extent the left piriform lobe, and reaching the left parietal cortex margins. The lesion caused a mild mass effect with a right‐sided midline shift, distortion of the left lateral ventricle, and decreased cerebrospinal fluid signal surrounding the cerebrum. The mass lesion was homogeneously hyperintense on T2‐weighted (T2W) and fluid‐attenuated inversion recovery (FLAIR) images compared to gray matter, and hypointense on T1‐weighted (T1W) images with no contrast enhancement (Figure 1). No signal void was observed on T2*‐gradient echo images and the mass was hyperintense on diffusion weighted imaging and on apparent diffusion coefficient map. Cerebrospinal fluid was collected from 9 dogs (but collection was not performed in the dog with the forebrain mass) and routine analysis (cell count, cytology, and protein concentration) was normal in all. One dog with normal MRI had inter‐ictal synchronized video‐electroencephalography (EEG; EBNeuro, Nemus II, Florence, Italy) performed. A bipolar montage was used to place electrodes as previously described.^12^ Initially, the dog was assessed unsedated while eating, and rare spikes were observed in both frontal and parietal cortex admixed with artifacts associated with blinking, eye movements, and muscular movements (Figure 2). Video‐EEG under sedation (dexmedetomidine constant rate infusion: 0.5‐2 μg/kg/h) was unremarkable (no spike and spike‐and‐wave complexes) and showed decreased background activity.

### 3.5 Diagnosis

Nine dogs had a presumptive diagnosis of idiopathic epilepsy (IE) and the dog with the forebrain mass had a presumptive diagnosis of glioma (Supplemental Table S1). Postmortem examination was not performed in any of the cases.

### 3.6 Treatment and outcome

Diet was changed after onset of STE in 5 dogs. Diets tried included other dry and canned commercial foods and a home‐made diet in 1 dog. No dog was reported to have clinically relevant improvement in seizure frequency or severity with diet change. Eating habits were changed in 8 dogs, and in 3 of them this change decreased their STE frequency (Supplemental Table S1 all 3 of these dogs had SRS). Slow feeder bowls, raised feeding bowls, decreased speed of food intake, and changing the frequency and size of the meal all were tried with no success in any of the dogs.

Nine dogs had antiepileptic treatment. Antiepileptic drugs used included phenobarbital (n = 7), levetiracetam (n = 7), potassium bromide (n = 3), imepitoin (n = 3), gabapentin (n = 1), and alprazolam (n = 1; Supplemental Table S1). All antiepileptic drugs were used at the recommended therapeutic dosage and serum concentrations were evaluated where appropriate. The dog with structural epilepsy was treated with a tapering dosage of prednisolone (from 1 mg/kg PO q24h to 0.5 mg/kg PO q48h within 3 weeks and continued at the lower dose) in addition to levetiracetam (20 mg/kg PO q8h; Supplemental Table S1).

After treatment, 1 dog became seizure‐free for the last 6 months of follow‐up, seizure severity improved in 3 dogs, and seizure frequency improved in 8, varying from an improvement of 25% to 90% in both STE and SRS frequency. Phenobarbital decreased seizure frequency in 6/7 dogs. Levetiracetam improved seizure frequency in 5/7 dogs, did not improve seizure frequency in 1 dog, and response could not be evaluated in another dog. Imepitoin did not improve seizure frequency in 2 treated dogs, and response was difficult to evaluate in another dog. Alprazolam also was ineffective in the dog it was used to treat. Peculiarities about the treatment can be found in Supplemental Table S1.

Appetite increased after onset of STE in 1 dog and remained unchanged in the remainder. Weight was reported to be stable in all but 1 dog, in which a slight increase in weight occurred. The dog with structural epilepsy was euthanized 1 month after the onset of STE. This dog had 5 STE, 3 before treatment, and 2 more a month later. Excluding this dog, the median follow‐up period after the first seizure was 24 months (range, 12‐96 months) and all were still alive at the time of writing.

## 4 DISCUSSION

We report for the first time STE in dogs and describe its clinical features. It seems that the prevalence in dogs might be as rare as it is in humans because despite an international call for cases through a widely publicized forum, only 11 cases were recruited. In all cases, STE represented >50% of seizures experience by the patient.

Eating is defined by the act of prehending food followed by chewing and swallowing.^13^ A clear definition of “eating epilepsy” cannot be found in the medical literature and previous studies in humans have included a range of epileptic seizures associated with eating, drinking, and food‐related stimuli.^9^, ^10^ Terminology used is inconsistent and terms such as eating‐related epilepsy have been suggested.^14^ More than 50% of seizures occurring during eating or within 30 minutes after a meal are some of the inclusion criteria used in the small number of studies describing large‐case series in humans.^10^, ^15^, ^16^

Eating epilepsy can be structural or genetic.^17^ Familial clusters in humans of presumed and confirmed idiopathic eating epilepsy exist in some geographical areas,^9^, ^15^, ^18^ and *MECP2* and *SYNGAP1* gene mutations have been associated with neurological syndromes, including STE among other clinical signs.^19^, ^20^ Interestingly, 44% (4/9) of the dogs diagnosed with IE in our study were retriever breeds, which are phylogenetically closely related to each other,^21^ and a breed predisposition may exist. None of the dogs in our study were reported to have other STE‐affected dogs in their pedigrees, but only 5 owners were in contact with the breeder or had knowledge of related dogs. There are far fewer reports of STE with structural etiology, but they represent 34% of patients with refractory eating epilepsy.^10^ Reported pathologies include congenital malformations, vascular abnormalities, postinfective lesions, mesial temporal sclerosis, gliotic pathology associated with postperinatal or post‐radiotherapy sequelae, and 2 neoplasms: an astrocytoma and a glioblastoma.^10^, ^22^, ^23^, ^24^, ^25^, ^26^, ^27^ One dog in our study had a presumptive diagnosis of glioma given the breed predisposition,^28^ location, and imaging characteristics^29^ described elsewhere.

Excluding the syndromes associated with mutations in the *MECP2* and *SYNGAP1* genes, the occurrence of STE is more common in the second decade of life and a male predisposition exists in both structural and genetic cases.^9^, ^10^, ^11^, ^15^, ^30^ The only 2 human patients described with gliomas were aged 14 and 44 years.^23^, ^24^ In contrast to what occurs in humans, we identified more female than male dogs and the age of onset (excluding the 1 case of structural STE) was >12 months and within the range expected for dogs with IE.^31^ The reasons for disparities are unknown, but results could be influenced by our low case numbers or may imply a different etiopathogenesis.

Similar to our findings, 33% of human patients with eating epilepsy have only STE,^15^ and this percentage increases to 49% in patients with eating epilepsy refractory to medical management.^10^ Seizures triggered by eating may be replaced by SRS and vice versa.^15^ Although STE are of variable semiology, the most common seizure type is focal complex seizures.^10^, ^11^, ^22^, ^32^ Simple focal, generalized tonic‐clonic, myoclonic, and atonic (ie, epileptic spasms manifesting as head drops) seizures all have been described,^10^, ^33^ and multiple seizure types can coexist.^10^, ^33^ Terms subclassifying focal epileptic seizures based on normal or impaired consciousness (simple or complex, respectively) are no longer used in human medicine and are discouraged in animals given the subjective interpretation of the experience.^1^ A focal onset followed by secondary generalization was suspected in all dogs, and, as occurs in humans, multiple seizure types coexisted. Classification can be challenging and there are inherent ambiguities in the recommended seizure classifications in clinical use.^34^ Other tools such as EEG^34^ or functional imaging^11^ may improve accuracy.

Given their predominantly focal origin, STE are thought to be localization‐related. Based on EEG findings in human patients, it traditionally has been believed that most originate from the temporo‐limbic region (temporal lobe epilepsy) and less frequently the extra‐temporal perisylvian region.^9^, ^11^, ^15^, ^22^, ^32^, ^35^, ^36^ The perysilvian region, defined as the region around the sylvian fissure or lateral sulcus which separates the temporal from the frontal and parietal lobes,^37^ contains the operculum which refers to portions of the frontal, parietal, and temporal lobes adjacent to this fissure and overlying the insula.^38^ Other localizations associated with STE in clinical patients include subcortical structures^23^ and the brainstem,^39^ which can be diffusely involved^10^, ^33^ and multifocal and asymmetric^10^ disorders also have been found in some patients, suggesting a multilobular network involving all cerebral lobes.^10^ Electroencephalography was only available in 1 of our cases, and as reported in humans during eating, the findings mainly included movement and muscle artifacts with some ictal discharges, which can obscure the recording making interpretation challenging.^9^ Up to 44% of human patients with STE have intra‐ictal findings that localize diffusely or of uncertain origin.^10^ Determining the exact ictal onset is difficult in patients with generalized epilepsy syndromes in which cortical‐subcortical interactions have been hypothesized as forerunners of epileptogenesis.^40^

Eating is a complex phenomenon that involves psychological, enteroceptive, proprioceptive, and sensory stimuli activating multiple areas in these multilobular networks, including subcortical structures such as hypothalamic nuclei and brainstem structures involved in the cephalic phase of digestion.^22^, ^23^, ^41^ Many factors such as sight,^42^ proprioceptive information from hands or arms,^18^, ^41^mastication,^42^, ^43^ swallowing, passage of food and esophageal stimulation,^44^ chemical factors,^23^, ^44^, ^45^ taste or smell,^19^ gastric distention or having a large meal,^18^, ^30^ the satisfied feeling associated with eating,^46^ and speed of food ingestion^30^ all can trigger seizures. The thought of food alone has been identified as a trigger in some cases. 11, 24 Environmental factors also might be involved, and some human patients only exhibit STE when at home.^30^ It is difficult to evaluate if eating location plays a role in eating epilepsy in dogs given that most dogs would rarely eat meals outside their home environment, and human patients have failed to identify any other environmental factors.^30^ A combination of several stimuli or the complete sequence of having a meal rather than the individual components might be necessary to trigger the seizure (collective sensory input of multimodal nature).^20^, ^46^ The pathophysiology is inevitably complex and although triggers are usually stereotypical for each patient, they may differ from one another.^20^ For example, although in some human patients feeding themselves triggered seizures, the same act was negligible in others.^33^ Interestingly, seizure frequency was decreased by hand feeding in 1 dog.

Together with genetic features, ethnic factors including specific eating habits (e.g. eating bulky meals rich in carbohydrates, mostly a rice‐based diet) have been proposed to explain the higher incidence in some geographical areas.^10^, ^15^ The carbohydrate and rice composition of the food eaten by the dogs in this study was unknown, but most ate a commercial diet. In experimental antimuscarinic‐induced convulsion mice models where food texture was investigated, seizures were triggered only by solid food intake (several minutes after ingestion),^47^ whereas in humans semi‐solid meals or consumption of liquids also can trigger seizures.^9^, ^10^, ^30^ Both canned and dry food triggered STE in most dogs with only 1 dog having seizures triggered by drinking. Seizures triggered by drinking are even rarer than STE with very few cases reported in humans.^9^, ^25^, ^30^ Interestingly, STE did not occur with liquified food in 1 dog.

Attempts have been made to correlate the type of stimuli and corresponding stimulated forebrain area with timing of seizure onset.^10^, ^22^, ^24^ Seizures triggered by eating in people typically occur shortly after beginning to eat and do not recur during the same meal.^10^, ^15^, ^32^ As in people, STE in dogs occurred typically at the beginning of the meal, which may imply similar triggering stimuli, but no conclusions can be made regarding the recurrence during the same meal given our low number of cases.

Evidence exists to support the existence of the multi‐lobular network of areas with a key role in eating epilepsy. Among others, the posterior cortex is thought to link visual and sensory inputs to the limbic‐opercularpathway,^10^ and orbitofrontal and temporo‐insulo‐opercular cortices show increased activity with visual food stimuli.^48^ The amygdala is frequently involved in masticatory movements during seizures^49^ and because of its low threshold for seizure activity, it is a likely target for the masticatory input that precipitates seizures evoked by eating.^30^ The involvement of the opercular region during seizures induced by mastication was identified using deep electrode implantations. Both structures are part of neuronal networks that process many different food stimuli, including taste, olfaction, and appetite modulation.^22^, ^24^, ^48^

Structural cases frequently involve the perisylvian region and more specifically the frontal operculum.^10^, ^11^, ^22^, ^24^, ^26^, ^36^, ^50^, ^51^ Pathologies in other areas including deep forebrain also have been found, reflecting the complex pathophysiology.^10^, ^23^, ^25^ One study found that in patients with refractory STE, MRI lesions were located in the posterior temporo‐parieto‐occipital cortex in 69% of patients, but the epileptiform discharges originated more frequently from the fronto‐centro‐temporal region.^10^ The human and canine cerebral cortex are different and the latter is poorly mapped. Most of the temporal cortex, some of the frontal cortex, and the boundaries of the temporal cortex with the frontal cortex and parietal cortex were affected in the dog with structural STE. Given the extension of the mass, the area corresponding to the perisylvian region in humans likely was affected. The key role of this region in eating epilepsy in humans has been identified using EEG,^11^ deep electrode implantation,^43^ functional MRI,^48^ and single‐photon emission computed tomography.^11^ Six of our dogs were scanned using low‐field MRI, which has a lower sensitivity compared to high‐field MRI and some subtle structural lesions might have been missed.^52^

Seizures triggered by eating can be prevented by modifying triggers in some cases.^2^, ^9^, ^14^, ^33^ For example, patients more sensitive to somatosensory or proprioceptive stimuli can alter the sensory characteristics of food. Characterizing specific triggering factors is difficult and frequently unsuccessful in human patients and therefore may pose an even bigger challenge in animals.^9^, ^14^, ^33^ Other simple things such as providing alerting stimuli during the meal to modify attention‐arousal coupling also can prevent seizures.^14^

When taken before meals, the benzodiazepine clobazam was shown to be effective in preventing STE in humans and although less effective, positive responses also were observed with other antiepileptic drugs (eg, phenobarbital) commonly used in dogs with epilepsy.^9^, ^15^, ^33^ Phenobarbital and levetiracetam seemed to be the most effective treatment for STE in our study, but they also were the most commonly used drugs. Given the rarity, the etiologic heterogeneity and the complex pathophysiology, recent studies evaluating treatment in humans are lacking. Studies show conflicting results regarding efficacy of antiepileptic drug treatment for STE.^9^, ^10^, ^15^ Prospective studies to assess the most effective antiepileptic drug for STE in dogs would be helpful, but given the rarity of the condition, recruiting cases may be difficult. As in humans, multimodal treatment including identifying and eliminating triggers when possible combined with the use of antiepileptic drugs and sometimes surgery may be the best approach.

Paroxysmal dyskinesias and related disorders can be difficult to distinguish from epileptic seizures. To avoid inclusion of cases with nonepileptic seizure‐like episodes, the suspected seizures had to be classified as such by 2 board‐certified neurologists based on the owner's description, video recordings, or both. The duration, presence of autonomic signs, and post‐ictal phase were consistent with epileptic seizures in all but 1 case. Furthermore, paroxysmal dyskinesias induced by eating are very rare in humans. Chewing‐induced dystonia has been described in just a few cases in humans with the dystonia remaining focal and limited to the face.^53^, ^54^, ^55^, ^56^ All of our cases showed generalized signs, but differentiation ideally would be achieved using intra‐ictal EEG monitoring.^57^, ^58^ Response to antiepileptic drugs is also supportive evidence that these episodes were seizures.

The main limitations of our study include its retrospective nature, the low number of cases, the lack of video recordings in 2 cases, the lack of EEG recordings in most cases, and the lack of postmortem examination in all cases. Our inclusion criteria and study design might have created a bias toward cases with easily recognizable seizure features. As previously discussed, STE is of variable semiology and features such as neck or trunk extension, unless accompanied by other clinical signs, might not have been recognized as epileptic seizures.^11^

In conclusion, STE occurs in dogs and can have etiological and semiological heterogeneity. Affected dogs may have STE only or it may occur in combination with SRS, and a predisposition in retrievers may exist. Given the complex pathophysiology, a thorough history is essential, and although frequently unsuccessful, the history should attempt to identify specific triggers. Electroencephalography often is challenging because of the presence of movement and muscles artifacts, but, like MRI, is helpful in the evaluation of eating epilepsy, which can be structural or idiopathic in dogs. Treatment should be considered on an individual basis and may need to vary according to etiology. A multimodal approach including identifying and eliminating triggers combined with pharmacological management may be necessary.

## CONFLICT OF INTEREST DECLARATION

Authors declare no conflict of interest.

## OFF‐LABEL ANTIMICROBIAL DECLARATION

Authors declare no off‐label use of antimicrobials.

## INSTITUTIONAL ANIMAL CARE AND USE COMMITTEE (IACUC) OR OTHER APPROVAL DECLARATION

Authors declare no IACUC or other approval was needed.

## HUMAN ETHICS APPROVAL DECLARATION

Authors declare human ethics approval was not needed for this study.

## Supporting information


**Appendix** **S1.** Supporting Information

**Table S1.**Table summarizing breed, gender, presence or absence of spontaneous recurrent seizures (SRS), age at onset of seizures triggered by eating (STE) and SRS, a description of the seizure, presence and duration of postictal signs, seizure semiology, diagnosis, pharmacological treatment and eating habits that reduced STE frequency

**Video S1.** Video of Case 10 illustrating the focal seizure starting after few food bites and evolving to become generalized and tonic‐clinic

**Video S2.** Video of Case 6 illustrating the seizure starting at the middle of the meal which quickly becomes generalized and tonic‐clonic
