# Supplementary material for: Epileptic seizures triggered by eating in dogs
Source: J Vet Intern Med. 2020 Apr 28;34(3):1231–8. doi: 10.1111/jvim.15773 (PMC7255664; doi:10.1111/jvim.15773)
Supplement: Supplementary file 1 — Appendix S1. Supporting Information [file JVIM-34-1231-s001.pdf]

Questionnaire to be completed by owner and board-certified neurologist.

Eating Epilepsy Study

Notes:

- If seizures are triggered mostly by drinking instead of eating please substitute eating by drinking and vice versa in the following questionnaire.
- **TBCV:** to be completed by veterinary surgeon

QUESTIONNAIRE:

General questions

1) Are seizures only related to eating or also spontaneous (spontaneous means there is no trigger)?
    - Yes/No
        - If No:
            - What percentage is spontaneous and what percentage related to eating? Meal _ _%, spontaneous _ _ %
                - Did this change over time? Yes/No
                    - If Yes, how?
            - Was the first witnessed seizure spontaneous or related to eating?
                - Spontaneous/Related to eating
2) Age at onset of eating seizures?
3) Age at presentation to the referring veterinarian?
4) Breed?
5) Gender?
6) Sexually intact, spayed, or castrated when then the first seizure occurred?
7) Is XX food orientated? Yes/No
8) Have you had difficulties training XX? Yes/No, If yes, please specify what with:
9) Any behavioral abnormalities in between seizures (not shortly before or after seizures)? Yes/No, If Yes, please specify:
10) Does XX suffer from: fear or anxiety, defensive aggression, and abnormal perception? Yes/No, If Yes, please specify:
11) Appetite:
    - How was XX's appetite at diagnosis: Poor, average, good
    - Has XX appetite changed since he/she started having eating seizures? Yes/No
        - If yes, how would you classify his current appetite?
        - If other please specify:
12) Any other dogs in the household?
    - Yes/No
13) Weight:
    - What was XX's weight at the time of diagnosis?
    - Has XX's weight changed since the onset of eating seizures?
        - Stable, increased, decreased
14) Does XX have any frequent vomiting or diarrhea? Yes/No
    - If yes, please specify which and frequency
15) Do you know any of XX's siblings? Yes/No
    - If Yes:

- Do any of them have seizures? Yes/No
- Have any of the seizures been triggered by eating? Yes/No

16) Was XX born by caesarean? Yes/No
17) Has XX had a previous severe head trauma?

Investigations and diagnosis

18) Physical exam findings? **TBCV**
19) Neurological exam findings? **TBCV**
20) Investigations and results? **TBCV**
    - Hematology (Performed: Yes/No, Normal: Yes/No, please specify abnormalities), Biochemistry (Performed: Yes/No, Normal: Yes/No, please specify abnormalities), Ammonia (Performed: Yes/No, Normal: Yes/No, please specify abnormalities), BAST (Performed: Yes/No, Normal: Yes/No, please specify abnormalities), Urinalysis (Performed: Yes/No, Normal: Yes/No, please specify abnormalities), Thoracic radiographs (Performed: Yes/No, Normal: Yes/No, please specify abnormalities), Abdominal ultrasound (Performed: Yes/No, Normal: Yes/No, please specify abnormalities), MRI (Performed: Yes/No, Normal: Yes/No, please specify abnormalities), CSF (Performed: Yes/No, Normal: Yes/No, please specify abnormalities), EEG (Performed: Yes/No, Normal: Yes/No, please specify abnormalities), Others: Please specify
21) What was XX's diagnosis? **TBCV**

Seizure related questions:

22) What was the seizure frequency at onset (before referral and starting antiepileptic drugs)?
    - Daily? Weekly? Monthly? Every 3-6 months? Yearly? Less than one a year?
    - If number of seizures and timing known please specify:
23) What was the seizure frequency in the first three months?
    - Daily? Weekly? Monthly? Every 3-6 months? Yearly? Less than one a year?
    - If number of seizures and timing known please specify:
24) Please select when do most eating seizures occur in relation to timing of food intake:
    - Before meal (i.e. visual stimulus, smell, example: has seizure just before having any bites of food when owner grabs eating bowl)
    - Beginning of meals (after a couple of bites)
    - Middle of meal (has eaten more than ½ of the meal)
    - After end of meal (no food left in the eating bowl)? If after meal, please specify how long after?
25) Do you have video footage? Yes/No
26) Do **eating seizures and spontaneous** seizures look the same?
    - Yes/No
        - If Yes, there is no need to answer question XX.
27) Do all **eating** seizures look the same?
    - Yes/No, If No, please answer question XX as many times as types of seizure XX has.
28) Do all **spontaneous** seizures look the same?
    - Yes/No, If No, please answer question XX as many times as types of seizure XX has

29) Seizure semiology (how do they look like) of the **eating** seizures:

- Do the seizures start with any twitching around the face or legs while standing (partial seizure)? Yes/No
- Do the seizures start with lifting of any limb (partial seizure)? Yes/No
- Does XX lay on his/her side and starts paddling (generalised clonic seizure)? Yes/No
- Does XX lay on his/her side and becomes rigid (generalised tonic seizure)? Yes/No
- Does XX lay on his/her side and becomes rigid but also paddles (generalised tonic-clonic seizure)? Yes/No
- Does XX stare into space for long periods of time and you are unable to distract him (absence seizure)? Yes/No
- Does XX have sudden and general loss of muscle tone causing him to collapse and be flaccid (atonic seizure)? Yes/No
- Does XX suffer from brisk jerking movements (myoclonic seizure) without falling to the side? Yes/No
- Any other important features? If so, please describe:
- **TBCV** please select type of seizure: Partial seizure? Generalised seizure? Tonic, clonic, tonic-clonic? Partial evolving to generalised? Myoclonic? Atonic? Absence?

30) Seizure semiology of the **spontaneous seizures** (if they occur):

- Do the seizures start with any twitching around the face or legs while standing (partial seizure)? Yes/No
- Do the seizures start with lifting of any limb (partial seizure)? Yes/No
- Does XX lay on his/her side and starts paddling (generalised clonic seizure)? Yes/No
- Does XX lay on his/her side and becomes rigid (generalised tonic seizure)? Yes/No
- Does XX lay on his/her side and becomes rigid but also paddles (generalised tonic-clonic seizure)? Yes/No
- Does XX stare into space for long periods of time and you are unable to distract him (absence seizure)? Yes/No
- Does XX have sudden and general loss of muscle tone causing him to collapse and be flaccid (atonic seizure)? Yes/No
- Does XX suffer from brisk jerking movements (myoclonic seizure) without falling to the side? Yes/No
- Any other important features? If so, please describe:
- **TBCV** please select type of seizure: Partial seizure? Generalised seizure? Tonic, clonic, tonic-clonic? Partial evolving to generalised? Myoclonic? Atonic? Absence?

31) Have you recognized any changes in behaviour just before the actual eating seizure episode (minutes or hours) (preictal signs)? **If different** for spontaneous versus eating seizures, **or** even the type of seizure please specify/answer this question multiple times.

- Yes/No; If yes, Which and how long does this behavior last?

32) Does XX take time to recover from the eating seizure (Post ictal signs)? **If different** for spontaneous versus eating seizures, **or** even the type of seizure please specify/answer this question multiple times.

- Yes/No he/she is normal straight away
    - If Yes, does he/she pace, circles, bangs into furniture, sleeps, is wobbly? Please specify. If others, please specify too.
    - How long do this sign last?

33) Does XX (**If different** for spontaneous versus eating seizures, **or** even the type of seizure please specify/answer this question multiple times)
    - Salivate during the seizure or very closely related to it? Yes/No; If very close related to it but not during the seizure please specify when.
    - Defecate during the seizure or very closely related to it? Yes/No; If very close related to it but not during the seizure please specify when.
    - Urinate during the seizure or very closely related to it? Yes/No; If very close related to it but not during the seizure please specify when.
34) Did cluster seizures (2 or more seizures within 24h) ever occur? Yes/No; If Yes:
    - How frequent are they?
    - Did this change over time? If yes, please specify
    - Did cluster seizures occur before or after starting medication, or both?
        - If both, did they increase/decrease/remained the same in frequency?
    - Were cluster seizures triggered by eating or seizures were spontaneous, or both?
    - Does XX cluster if he/she attempts finishing the meal he/she initially started eating? Or at another meal later in the day?
35) Did status epilepticus ever occur (a seizure lasting more than 5 minutes or not recovering between 2 seizures that occurred in less than 24 hours)? Yes/No
    - If yes which of the following occurred: 1) a seizure lasting more than 5 minutes, 2) not recovering between 2 seizures that occurred in less than 24 hours
36) Diet:
    - What food was XX's food at onset of eating seizures?
    - Food consistency at onset of eating seizures: Dry? Wet? Both?
    - Had any recent diet change been made at onset of eating seizures? Yes/No
    - Did the diet contain rice? What was the % of carbohydrates? This is usually specified in the food package

Attempting to identify triggers:

37) Is food always available for XX: Yes/No; If No, how many times is XX fed per day?
38) Does XX get treats? Yes/No
    - Have treats ever triggered a seizure? Yes/No; If yes please quantify selecting one of the following: once; just a few times of the many seizures, 25%, 50%, 75% or 100% of all seizures are triggered by eating treats.
39) Speed:
    - How quickly does XX eat? Fast (eats quickly as if his food is going to be stolen), Slowly (takes a few bites every now and then and comes back to the bowl several times before finishing the meal), Not fast neither slow (keeps eating at steady speed but not quickly) Variable speed: sometimes eats slowly, others fast.
    - Do seizures occur more commonly when eating: Fast? Slowly? Not fast neither slow? Does not make a difference? I don't know.
40) Which food consistency triggers eating seizures: Dry? Wet? Both? I have just tried dry food or I have just tried wet food.
41) Food quantity, please select: Big meals trigger eating seizures; Small meals trigger eating seizures; The size of the meal does not seem to make a difference; I don't know if the size of the meal makes a difference

42) Timing, please select: Eating early triggers eating seizures? Eating late triggers eating seizures? Neither eating late or eating early triggers eating seizure? I don't know if timing has an effect
43) Temperature of food, please select: Eating hot food triggers eating seizures? Eating cold food triggers eating seizures? Eating room temperature food triggers eating seizures? All trigger eating seizures? Never tried giving XX hot/cold food?
44) Relation of eating seizures to type of meal. Eating seizures occur mostly at: Breakfast? Lunch? Dinner? There is no relation to meal of the day?
45) Does the excitement caused by getting food ready precipitate seizures? Yes/No
46) Does seeing food precipitate seizures? Yes/No/I don't know
47) Does smelling food precipitate seizures? Yes (you have tried to just let him smell food and that has occasionally been enough to precipitate eating seizures)/No/I don't know
48) Has drinking ever triggered a seizure? Yes/No; If yes, how frequently? Once; just a few times of the many seizures 25%, 50%, 75% or 100% of all seizures
49) Have you identified any other trigger for XX's seizures (related or unrelated to eating)? Yes/No; If Yes, please specify:
50) Date at completion of the questionnaire?

Treatment: Diet

51) Was diet changed at some point during treatment? Yes/No; If yes:
    - Was it changed to dry, wet or both?
    - Did the new diet contain rice? What was the % of carbohydrates?
    - Any improvement? Yes/No
        - Did XX become seizure free? Yes/No
        - If initial clusters, do clusters still occur? Yes/No
        - Was there a reduction in seizure severity? Yes/No
        - Was there a reduction in seizure frequency? Yes/No
            - If Yes:
                - What percentage?
                - Was the reduction in eating seizures, spontaneous ones or both?
            - If No:
                - The frequency remained the same or increased?
                    - Was the increase in eating seizures, spontaneous ones or both types of seizure?
                    - If remained the same, did the proportion of eating seizures and spontaneous ones change?

Treatment: Antiepileptic's

52) Were antiepileptic's used? Yes/No
    - If Yes:
        - Name of antiepileptic (if multiple and known please specify for each for each of the following questions)
        - Did XX become seizure free? Yes/No
        - If initial clusters, do clusters still occur? Yes/No
        - Was there a reduction in seizure severity? Yes/No
        - Was there a reduction in seizure frequency? Yes/No
            - If Yes:

- What percentage?
- Was the reduction in eating seizures, spontaneous ones or both?
- If No:
  - The frequency remained the same or increased?
    - Was the increase in eating seizures, spontaneous ones or both types of seizure?
    - If remained the same, did the proportion of eating seizures and spontaneous seizures change?

Treatment: Eating habits

53) Where any eating habits changed? Yes/No
- If Yes:
  - Any of the following: raised bowl? Feeding frequency? Size of meal?
  - If others please specify:
  - Response (if known to which habit changed please specify):
    - Did XX become seizure free? Yes/No
    - If initial clusters, do clusters still occur? Yes/No
    - Was there a reduction in seizure severity? Yes/No
    - Was there a reduction in seizure frequency? Yes/No
      - If Yes:
        - What percentage?
        - Was the reduction in eating seizures, spontaneous ones or both?
      - If No:
        - The frequency remained the same or increased?
          - Was the increase in eating seizures, spontaneous ones or both types of seizure?
          - If remained the same, did the proportion of eating seizures and spontaneous seizures change?
