# Supplementary material for: Epileptic seizures triggered by eating in dogs
Source: J Vet Intern Med. 2020 Apr 28;34(3):1231–8. doi: 10.1111/jvim.15773 (PMC7255664; doi:10.1111/jvim.15773)
Supplement: Supplementary file 2 — Table S1.Table summarizing breed, gender, presence or absence of spontaneous recurrent seizures (SRS), age at onset of seizures triggered by eating (STE) and SRS, a description of the seizure, presence and duration of postictal signs, seizure semiology, diagnosis, pharmacological treatment and eating habits that reduced STE frequency [file JVIM-34-1231-s002.pdf]

Supplemental Table 1. Table summarizing breed, gender, presence or absence of spontaneous recurrent seizures (SRS), age at onset of seizures triggered by eating (STE) and SRS, a description of the seizure, presence and duration of postictal signs, seizure semiology, diagnosis, pharmacological treatment and eating habits that reduced STE frequency.

Case number	Breed	Gender	ES alone or ES and SS	Age at onset of ES/SS (months)	Seizure description based on questionnaire and video footage	Post-ictal signs and duration	Seizures semiology	Diagnosis	Pharmacological treatment attempted	Eating habits changed that resulted in reduced ES frequency
1	Golden Retriever	ME	ES and SS	36/42	Contorts head to one side, opens mouth and keeps it open for several seconds, lifts legs before falling to lateral recumbence and then becoming a GTCS.	Y/up to 20 min	Partial evolving to GTCS	IE	Phenobarbitone	Preparing food away/reducing excitement
2	Curly Coated Retriever	ME	Eating and drinking seizures	14	Has difficulty ambulating forward, facial twitching, sometimes walks backwards, lifts a thoracic limb (mainly left in video footage) intermittently, gets mildly kyphotic, looks scared, confused, has small jerks and generalised tremors, only turns left in video footage. Does not fall into lateral recumbence	Y/up to 120 min	Partial evolving to generalised	IE	Phenobarbitone	N/A
3	Bichon Frise	FN	ES and SS	36/36	Facial or leg twitching before becoming a GTCS. After starting treatment seizures became myoclonic	Y/for 10 min after GTCS but is normal straight after myoclonic seizures	Partial evolving to GTCS and myoclonic seizures	IE	Phenobarbitone Levetiracetam	N/A
4	Jack Russell Terrier	FN	ES	36	Facial or leg twitching and lifting a thoracic limb before becoming a GTCS.	Y/up to 120 min	Partial seizures evolving to GTCS	IE	N/A	N/A
5	Flat Coated Retriever	ME	ES and SS	15/15	Facial twitching, lifting and twitching of a thoracic limb, falling to lateral recumbence and becoming GTCS but not always losing consciousness. Sometimes wants to keep eating	Y/normally 30 min but lasting up to 120 min	Partial seizures sometimes evolving to GTCS	IE	Phenobarbitone Levetiracetam Potassium bromide	N/A
6	Poodle	FN	ES and SS	12/12	Vigorous head shacking, facial or leg twitching that quickly progresses to GTCS	Y/usually for 10 min	Partial seizures evolving to GTCS	IE	Phenobarbitone Levetiracetam Potassium bromide Imepitoin	N/A
7	English Staffordshire Bull Terrier	MN	ES and SS	24/24	Mouth chattering, turns head, and becomes GTCS	Y/unknown length of time	Partial seizures evolving to GTCS	IE	Phenobarbitone Levetiracetam Imepitoin Alprazolam Gabapentin	Hand feeding
8	Golden Retriever	FE	ES	27	Facial twitching and opening mouth and progresses to GTCS	Y/up to 120 min	Partial seizures evolving to GTCS	IE	Levetiracetam	N/A
9	Border Collie	FN	ES and SS	69/30	Chattering, opens mouths and falls to lateral recumbence, sometimes evolving to GTCS	Y/15-20min	Partial seizures sometimes evolving to GTCS	IE	Phenobarbitone Levetiracetam Potassium bromide Imepitoin	Providing liquidised food
10	Border Terrier	FN	ES	120	Facial twitching and myoclonic jerks evolving to GCTS	N	Partial seizures evolving to GTCS	Structural epilepsy	Levetiracetam Prednisolone	N/A

ES: eating seizures; SS: spontaneous seizures; IE: idiopathic epilepsy; Y: Yes; N: No; GTCS: generalised tonic-clonic seizure; Min: minutes; FN: female neutered; FE: female entire; MN: male neutered; ME: male entire, N/A: not applicable
